# The variation in the health status of immigrants and Italians during the global crisis and the role of socioeconomic factors

**DOI:** 10.1186/s12939-017-0596-9

**Published:** 2017-06-12

**Authors:** Alessio Petrelli, Anteo Di Napoli, Alessandra Rossi, Gianfranco Costanzo, Concetta Mirisola, Lidia Gargiulo

**Affiliations:** 1 National Institute for Health, Migration and Poverty (INMP), Via San Gallicano, 25 a, 00153 Rome, Italy; 2 National Institute of Statistics (ISTAT), Viale Liegi, 13, 00198 Rome, Italy

**Keywords:** Immigrants, Socioeconomic, Health inequalities, Economic global crisis, Italy, Survey

## Abstract

**Background:**

The effects of the recent global economic and financial crisis especially affected the most vulnerable social groups. Objective of the study was to investigate variation of self-perceived health status in Italians and immigrants during the economic global crisis, focusing on demographic and socioeconomic factors.

**Methods:**

Through a cross-sectional design we analyzed the national sample of multipurpose surveys “Health conditions and use of health services” (2005 and 2013) conducted by the Italian National Institute of Statistics (ISTAT). Physical Component Summary (PCS) and Mental Component Summary (MCS) scores, derived from SF-12 questionnaire, were assumed as study outcome, dichotomizing variables distribution at 1^st^ quartile. Prevalence rate ratios (PRR) were estimated through log-binomial regression models, stratified by citizenship and gender, evaluating the association between PCS and MCS with surveys’ year, adjusting for age, educational level, employment status, self-perceived economic resources, smoking habits, body mass index.

**Results:**

From 2005 to 2013 the proportion of people not employed or reporting scarce/insufficient economic resources increased, especially among men, in particular immigrants. Compared with 2005 we observed in 2013 among Italians a significant lower probability of worse PCS (PRR = 0.96 both for males and females), while no differences were observed among immigrants; a higher probability of worse MCS was observed, particularly among men (Italians: PRR = 1.26;95%CI:1.22–1.29; immigrants: PRR = 1.19;95%CI:1.03–1.38). Self-perceived scarce/insufficient economic resources were strongly and significantly associated with worse PCS and MCS for all subgroups. Lower educational level was strongly associated with worse PCS in Italians and slightly associated with worse MCS for all subgroups. Being not employed was associated with worse health status, especially mental health among men.

**Conclusions:**

Our findings support the hypothesis that economic global crisis could have negatively affected health status, particularly mental health, of Italians and immigrants. Furthermore, results suggest socioeconomic inequalities increase, in economic resources availability dimension. In a context of public health resources’ limitation due to financial crisis, policy decision makers and health service managers must face the challenge of equity in health.

## Background

Migration flows from developing countries towards Europe and Italy in particular, both for economic reasons and to flee war and persecution, has increased in the past few years. In particular, in the period 2013–2015, about half million people reached the Italian coasts: out of them, about 40% applied for international protection [1].

In Italy, the most recent estimates from the Italian National Institute of Statistics indicate that the number of resident immigrants has doubled in the last decade, from 2.4 million people (4.1% of the resident population) in 2005 to 5 million (8.2% of the resident population) in 2015 [2], of which 52.7% females. The average age is 31.1 for males and 34.1 for females. Out of them, almost 3.5 million (70.3%) are non-EU citizens: 22.7% from European countries, 20.5% from Africa, 19% from Asia and about 8% from the other continents [3]. The most represented immigrant groups in Italy are from Romania (22.6%), Albania (9.8%), Morocco (9.0%), China (5.3%) and Ukraine (4.5%).

First generation immigrants generally have better health conditions than the resident population. Two factors can act in determining this observation: the ‘healthy migrant effect’, the natural selection determining higher tendency to migrate in younger or healthier people [4, 5], and the selection in the return to the country of origin (salmon bias) [6, 7]. The relative role of this selective strength has not been clarified, while recent evidence from Europe tend to support the healthy migrant effect [8–11]. However, this health advantage tends to decrease once in the host country [12], socioeconomic difficulties that immigrants usually experience, accentuated by greater limitation in social mobility and discrimination [13], and also for assimilation of most deprived population lifestyle.

The effects of the recent global economic and financial crisis especially affected the most vulnerable social groups [14], to which most of immigrants belong. Between 2008 and 2013, employment significantly decreased, especially amongst immigrants (−9% compared to - 2.5% amongst Italians), contributing to deepen socioeconomic inequalities [15].

Many studies showed that the adverse effects of the crisis on population’s health, although with different intensity and elasticity, mostly affected non-developed countries, where welfare systems are weaker, and disadvantaged populations, in which the major social health risks, such as unemployment and poverty, are generally present [16, 17]. In this sense, the economic crisis emphasized socioeconomic health inequalities [18, 19]. Moreover, austerity policies, adopted in many countries to face the crisis, further exposed these particularly vulnerable populations to such disadvantages [20].

It is widely recognised that conditions of greater deprivation can negatively influence health outcomes [21, 22], both in physical [23–25] and mental [26] terms. There is significant evidence supporting an association between loss of employment and economic resources and mental health worsening [27, 28]. Negative effects on mental health were particularly observed among immigrants, who are more greatly affected by the increase of unemployment [19].

In Italy, some studies investigated the health of immigrants [29–31]; however, as far as we know, no evidence are available about the health status of Italians and immigrants during the recent global economic crisis on.

The National Institute for Health, Migration and Poverty (INMP) monitors the health status of disadvantaged population groups and perform scientific research through manages its National Epidemiologic Observatory on Immigrants and Poverty (OENIP). INMP started an institutional collaboration with the Italian National Institute of Statistics (ISTAT)., ISTAT carried out multipurpose surveys aimed at evaluating Italian and immigrant resident people’s health and use of health services in 2005 and 2013, so just before and after global economic crisis. In this sense we could indirectly observe the potential effect of the crisis on the health status of the population.

The aim of the present study was to compare the variation in the health status of Italians and immigrants between 2005 and 2013 by evaluating the self-perception of physical and mental health, and exploring the role of demographic and socioeconomic factors.

## Methods

This study was based on data of the multipurpose survey “Health conditions and use of health services” of 2013 and 2005 carried out by ISTAT on a representative sample of people residing in Italy. Two stage sampling method was used: in the first stage municipalities were firstly stratified into large cities and small towns and villages. All the large cities were included, while small towns and villages were selected with probability proportional to their size. In the second stage, families were selected with random criterion from the municipal registry lists. All the components of each selected families were included in the sample. The survey collected information on health conditions, health determinants and use of health services.

In both editions, data collection was carried out in four different surveys, conducted every three months. This method was aimed at taking into account the seasonal effects affecting health. Information was collected through PAPI (Paper and Pencil Interview) interviews to each member of the *de facto* family, conducted at the family home by interviewers trained by ISTAT. Some information was collected through self-administered individual questionnaires [32].

The sample is composed of families residing in Italy (2013 *n* = 48,811; 2005 *n* = 50,474) and their members (2013 *n* = 119,073; 2005 *n* = 128,041). The 2013 survey was conducted from July 2012 to June 2013, and the 2005 edition from October 2004 to September 2005.

As present study refers to working age people, we only took into account the sub-sample of people aged between 18 and 64 (in 2013 *n* = 72.476 and in 2005 *n* = 80.661), which represents a population of 37,290,440 people resident in Italy (33,900,000 Italians and 3,390,440 immigrants) in 2013, and of 36,852,745 (35,040,000 Italians and 1,812,745 immigrants) in 2005.

Immigrant status was defined using information about citizenship, dichotomized in two categories: Italian and foreigner.

In order to measure self-perception of health, two health status indexes were used: Physical Component Summary (PCS) and Mental Component Summary (MCS), both derived from the Short Form Health Survey (SF-12) and largely used in a number of empirical studies on European populations. SF-12 contains twelve questions on eight different dimensions related to health: physical activity, work limitations for health reasons, emotional state, physical pain, self-perceived general health, vitality, social activities and mental health [33].

Very low PCS values indicate poor physical health, limited self-care and physical, social and personal activity, serious physical pain. On the other hand, very high PCS values indicate excellent physical health without physical limitations, disability and reduction of general wellbeing and high vitality. We considered as status of worse physical health a PCS value up to the 1^st^ quartile of its total distribution in the population studied in the two survey editions. The cut-off was PCS value equal to 52.

Very low MCS values indicate poor mental health with frequent psychological discomfort, significant social and personal disability due to emotional problems. On the other hand, very high MCS levels indicate excellent mental health with frequent positive psychological attitude, absence of psychological discomfort and of limitations to social and personal activities due to emotional problems. We considered as worse physical health status a MCS value up to the 1^st^ quartile of its total distribution in the population studied in the two survey editions. The cut-off was MCS value equal to 46.

The PCS and MCS distribution at 1^st^ quartile cannot be interpreted as the prevalence of bad health status and does not have any diagnostic implication on the health status.

We estimated prevalence rate ratios (PRR) by using log-binomial regression models and dichotomised MCS and PCS as outcome variables to evaluate the association with survey’s edition (2005/2013), age group (18–34, 35–49, 50–64), level of education (high, medium, low), employment (yes/not), self-perceived economic resources (excellent/adequate, scarce/insufficient), smoking habits (never smoked, former smoker, smoker), body mass index (normal weight, underweight, overweight/obese). Each regression model was stratified by gender and citizenship (Italians/immigrants).

The socioeconomic covariates were progressively included in the models. Following interactions were also tested: a) socioeconomic covariates and citizenship; b) survey’s edition and citizenship; c) survey’s edition and socioeconomic factors. All analyses were performed using not weighted sample data. Statistical analysis was performed with SAS System 9.3.

## Results

Table 1 summarises some socio-demographic, clinical and lifestyle characteristics of Italians and immigrants respectively, stratified by gender, and compares the results of the 2013 and the 2005 survey. Out of total residents aged 18–64, immigrants in the sample are more than doubled, from 3.2% in 2005 to 7.1% in 2013. In relative terms, the immigrant population in the age group 50–64 increased from 11.2% in 2005 to 18.1% in 2013 (relative increase 60.9%), whereas Italians from 30.8 to 35.3% (relative increase 14.4%). This apparently faster ageing of foreigners is attributable to the fact that, on average, those who migrated more recently were older.

**Table 1:** Comparison between characteristics of population and year, by citizenship and gender

Variable		Italians					Immigrants				
		2005		2013			2005		2013		
		*n*	%	*n*	%	*p* value	n	%	*n*	%	*p* value
Men											
n		38,581		33,355			1,203		2,302		
PCS	I quartile	9,756	25.3	8,367	25.1	n.s.	220	18.3	465	20.2	n.s.
MCS	I quartile	7,277	18.9	8,513	25.5	<.0001	216	18.0	551	23.9	<.0001
Age	18–34	12,985	33.7	9,512	28.5	<.0001	554	46.1	924	40.1	<.0001
	35–49	13,833	35.9	12,227	36.7		540	44.9	1,028	44.7	
	50–64	11,763	30.5	11,616	34.8		109	9.1	350	15.2	
Educational level	High	16,551	42.9	17,014	51.0	<.0001	386	32.1	823	35.8	<.0001
	Medium	16,779	43.5	14,161	42.5		584	48.6	1,213	52.7	
	Low	5,251	13.6	2,180	6.5		233	19.4	266	11.6	
Occupational status	Employed	28,524	73.9	22,541	67.6	<.0001	1,074	89.3	1,704	74.0	<.0001
	Unemployed	10,057	26.1	10,814	32.4		129	10.7	598	26.0	
Self-perceived economic resources	Excellent/adequate	27,840	72.2	20,978	62.9	<.0001	525	43.6	817	35.5	<.0001
	Scarce/insufficient	10,741	27.8	12,377	37.1		678	56.4	1,485	64.5	
Smoking habits	Never smoked	16,403	42.5	14,277	42.8	n.s.	592	49.2	1,135	49.3	n.s.
	Former smoker	9,797	25.4	8,553	25.6		206	17.1	419	18.2	
	Smoker	12,381	32.1	10,525	31.6		405	33.7	748	32.5	
BMI	Normal weight	18,503	48.0	15,605	46.8	<0.001	644	53.5	1,148	49.9	n.s.
	Underweight	343	0.9	255	0.8		15	1.3	21	0.9	
	Overweight/obese	19,735	51.2	17,495	52.5		544	45.2	1,133	49.2	
Women											
n		39,469		33,940			1,408		2,879		
PCS	I quartile	12,585	31.9	10,533	31.0	<0.01	354	25.1	795	27.6	n.s.
MCS	I quartile	11,046	28.0	11,228	33.1	<.0001	311	22.1	786	27.3	<0.001
Age	18–34	12,850	32.6	9,077	26.7	<.0001	644	45.7	1,095	38.0	<.0001
	35–49	14,332	36.3	12,751	37.6		580	41.2	1,199	41.7	
	50–64	12,287	31.1	12,112	35.7		184	13.1	585	20.3	
Educational level	High	17,680	44.8	18,408	54.2	<.0001	571	40.6	1,319	45.8	<.0001
	Medium	14,601	37.0	12,369	36.4		608	43.2	1,258	43.7	
	Low	7,188	18.2	3,163	9.3		229	16.3	302	10.5	
Occupational status	Employed	18,949	48.0	15,914	46.9	<0.01	747	53.1	1,425	49.5	<0.05
	Unemployed	20,520	52.0	18,026	53.1		661	47.0	1,454	50.5	
Self-perceived economic resources	Excellent/adequate	27,911	70.7	21,109	62.2	<.0001	746	53.0	1,133	39.4	<.0001
	Scarce/insufficient	11,558	29.3	12,831	37.8		662	47.0	1,746	60.7	
Smoking habits	Never smoked	25,208	63.9	21,239	62.6	<.0001	981	69.7	2,058	71.5	n.s.
	Former smoker	6,197	15.7	5,931	17.5		180	12.8	354	12.3	
	Smoker	8,064	20.4	6,770	20.0		247	17.5	467	16.2	
BMI	Normal weight	24,818	62.9	21,083	62.1	<0.01	922	65.5	1,730	60.1	<.0001
	Underweight	2,561	6.5	2,128	6.3		95	6.8	142	4.9	
	Overweight/obese	12,090	30.6	10,729	31.6		391	27.8	1,007	35.0	

During the study period, socioeconomic conditions worsened, especially among men, particularly immigrants. The number of not employed men in the considered sample is much more increased among immigrants (from 10.7 to 26.0%, relative increase of 142.4%) than Italians (from 26.1 to 32.4%, relative increase of 24.4%). The increase of not employed people was lower amongst women, in relative terms higher amongst immigrants than Italians (7.6% vs 2.2%).

The percentage of immigrants reporting scarce/insufficient economic resources was much higher than Italians, both in 2005 (51.3% vs 28.6%) and 2013 (62.4% vs 37.5%), even though in relative terms the increase was higher amongst Italians (31.1% vs 21.5% for immigrants).

It is to be noted how this worsening in socioeconomic conditions took place notwithstanding the increase of people with higher educational level both among immigrants (from 36.7 to 41.3%, relative increase of 12.8%) and Italians (from 43.9 to 52.6%, relative increase of 20.0%).

There was a slight increase in overweight and obesity amongst the Italians between 2005–2013 (from 40.8 to 41.9%, relative increase of 2.8%). Whereas such increase was more significant amongst immigrants, both men (from 45.2 to 49.2%) and women (from 27.8 to 35%), amongst whom a higher variation in relative terms was reported (26.0% vs 8.8% amongst men).

The percentage of smokers slightly diminished, especially among immigrants, who smoked less than Italians in 2013 (23.5% vs 25.7%).

**Fig. 1:**
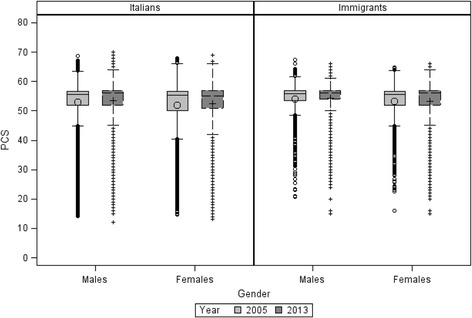
Distribution of PCS by gender and year

Figure 1 shows the distribution of PCS for Italian and immigrant men and women in 2005 and 2013. Among men, we observed similar median PCS values both in immigrants and Italians in 2005 (55.9 vs 55.5) and in 2013 (both 56.0). Among women, median PCS values were similar in 2005 (55.7 vs 55.3) and slightly higher for immigrants than Italians in 2013 (56.0 vs 55.0).

**Fig. 2:**
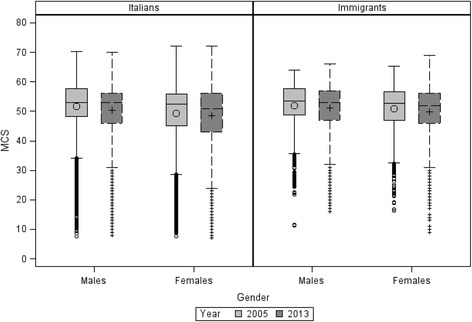
Distribution of MCS by gender and year

Figure 2 shows the distribution of MCS for Italian and immigrant men and women, in 2005 and 2013. Among men, median MCS values were similar for immigrants and Italians (53.6 vs 53.0). Among women, median MCS values decreased from 2005 to 2013 both for Italians (52.6 vs 51.0) and immigrants (52.8 vs 52.0).

Table 2 shows the results of the multivariate log-binomial model analysing factors associated with low PCS levels (PCS value ≤ 1^st^ quartile), stratified by citizenship and gender.

**Table 2:** Factors associated to the probability to have worse self-perceived physical health (I quartile PCS), by gender and citizenship

Variable		Men						Women					
		Italians			Immigrants			Italians			Immigrants		
		PRR	IC 95%		PRR	IC 95%		PRR	IC 95%		PRR	IC 95%	
Year	2005	1	-	-	1	-	-	1	-	-	1	-	-
	2013	0.96	0.94	0.99	1.03	0.89	1.19	0.96	0.94	0.98	1.00	0.90	1.12
Age	18–34	1	-	-	1	-	-	1	-	-	1	-	-
	35–49	1.59	1.52	1.65	1.36	1.15	1.60	1.50	1.45	1.56	1.20	1.07	1.36
	50–64	2.24	2.15	2.33	2.18	1.80	2.63	2.22	2.14	2.30	1.68	1.47	1.93
Educational level	High	1	-	-	1	-	-	1	-	-	1	-	-
	Medium	1.24	1.21	1.28	0.94	0.81	1.09	1.16	1.13	1.19	0.98	0.88	1.09
	Low	1.45	1.40	1.50	0.98	0.80	1.20	1.34	1.30	1.39	1.18	1.03	1.36
Occupational status	Employed	1	-	-	1	-	-	1	-	-	1	-	-
	Not employed	1.21	1.18	1.24	0.99	0.84	1.17	1.05	1.03	1.07	1.10	1.00	1.22
Self-perceived economic resources	Excellent/adequate	1	-	-	1	-	-	1	-	-	1	-	-
	Scarce/insufficient	1.24	1.21	1.27	1.27	1.10	1.47	1.18	1.16	1.20	1.17	1.06	1.30
Smoking habits	Never smoked	1	-	-	1	-	-	1	-	-	1	-	-
	Former smoker	1.28	1.24	1.32	1.38	1.17	1.64	1.19	1.16	1.22	1.30	1.14	1.48
	Smoker	1.10	1.06	1.13	1.24	1.07	1.45	1.09	1.06	1.12	1.16	1.02	1.32
BMI	Normal weight	1	-	-	1	-	-	1	-	-	1	-	-
	Underweight	1.35	1.19	1.54	1.04	0.46	2.35	1.02	0.97	1.08	0.97	0.75	1.26
	Overweight/obese	1.13	1.10	1.16	1.18	1.02	1.35	1.25	1.22	1.28	1.29	1.17	1.43

Compared to 2005, in 2013 we observed lower PRR of worse self-perceived physical health among Italians, while no differences were observed among immigrants, both for men and women. No interaction was observed between year and citizenship. It was observed that PRR increases with age in all considered groups. Furthermore, we observed a direct linear trend between education level and PCS among Italians (*p* < 0.05). An association between low educational level and a lower PCS was only observed in immigrant women. Italian and immigrant women showed a higher probability of PCS < =1^st^ quartile if not employed. Self-perceived scarce/insufficient economic resources are significantly associated with lower PCS levels in all the considered groups. All socioeconomic covariates appear to be independent factors for PCS (data not shown). Being a smoker or former-smoker is a condition independently associated with worse self-perceived physical health. Being overweight or obese is associated with higher probability of a worse self-perceived physical health, stronger among women than men, whereas an association with being underweight was only observed amongst Italian men.

Table 3 shows the results of the multivariate log-binomial model analysing factors associated with low MCS levels (MCS value ≤ 1^st^ quartile), stratified by citizenship and gender.

**Table 3:** Factors associated to the probability to have worse self-perceived mental health (I quartile MCS), by gender and citizenship

		Men						Women					
		Italians			Immigrants			Italians			Immigrants		
		PRR	IC 95%		PRR	IC 95%		PRR	IC 95%		PRR	IC 95%	
Year	2005	1	-	-	1	-	-	1	-	-	1	-	-
	2013	1.26	1.22	1.29	1.19	1.03	1.38	1.13	1.11	1.16	1.17	1.05	1.32
Age	18–34	1	-	-	1	-	-	1	-	-	1	-	-
	35–49	1.40	1.35	1.46	1.16	1.00	1.33	1.16	1.12	1.19	1.19	1.06	1.34
	50–64	1.51	1.45	1.57	1.42	1.19	1.70	1.29	1.25	1.33	1.23	1.07	1.43
Educational level	High	1	-	-	1	-	-	1	-	-	1	-	-
	Medium	0.98	0.95	1.01	1.02	0.88	1.17	0.98	0.95	1.00	0.95	0.86	1.07
	Low	1.08	1.03	1.13	1.17	0.98	1.41	1.10	1.07	1.14	1.17	1.01	1.37
Occupational status	Employed	1	-	-	1	-	-	1	-	-	1	-	-
	Not employed	1.32	1.28	1.36	1.54	1.35	1.76	1.04	1.02	1.07	0.98	0.88	1.08
Self-perceived economic resources	Excellent/adequate	1	-	-	1	-	-	1	-	-	1	-	-
	Scarce/insufficient	1.56	1.52	1.61	1.43	1.24	1.65	1.44	1.41	1.47	1.65	1.47	1.85
Smoking habits	Never smoked	1	-	-	1	-	-	1	-	-	1	-	-
	Former smoker	1.19	1.15	1.23	1.32	1.13	1.55	1.18	1.14	1.21	1.42	1.24	1.63
	Smoker	1.24	1.20	1.29	1.20	1.04	1.38	1.26	1.23	1.29	1.30	1.14	1.47
BMI	Normal weight	1	-	-	1	-	-	1	-	-	1	-	-
	Underweight	1.31	1.16	1.49	0.77	0.38	1.59	1.09	1.04	1.14	1.22	0.99	1.51
	Overweight/obese	1.01	0.98	1.04	1.09	0.96	1.24	1.09	1.06	1.11	0.95	0.85	1.06

Compared with 2005, in 2013 we observed lower PRR of self-perceived mental health for all the considered groups, particularly among men. No interaction was observed between year and citizenship. Higher probability of lower self-perceived mental health was observed with age increasing in all considered groups. We observed an association between low educational level and higher probability of MCS < =1^st^ quartile. Not employed men presented a higher PRR of mental health status, while no association was found for immigrant women. Self-perceived scarce/insufficient economic resources are the strongest predictor of MCS < =1^st^ quartile: all the considered groups show a probability higher than 40%. All socioeconomic covariates appear to be independent factors for MCS (data not shown). Being a smoker or former-smoker is a condition independently associated with worse self-perceived mental health. Not being in normal weight is associated with higher probability of a worse self-perceived mental health only among Italian women, while an association with being underweight was only observed amongst Italian men.

## Discussion

The results of our study, based on a representative sample of population residing in Italy, showed a worsening in the mental health status between 2005 and 2013, both among Italians and immigrants, also taking into account the effect of age, socioeconomic and lifestyle factors, while we did not observe significant differences in physical health status in the same period.

In this context, our findings support the hypothesis that the worsening of socioeconomic conditions observed during this period could have contributed to mental health decline [19], particularly amongst immigrants [34], as our data refer to years just before and after global economic crisis. Conversely to what was observed in Spain, a country with similar socioeconomic characteristics as Italy [35], we did not find any increase in differences in poor self-perceived mental health amongst immigrant women compared with natives.

Regarding physical health, a slight improvement was only observed among Italians. According to some studies, in developed countries the economic crisis can determine pro-cyclical effects on health, at least over the short term [36], with a protective effect on mortality – with the exception of suicides [37] – especially in countries with advanced welfare systems able to attenuate its negative effects [16].

Moreover, immigrants in Italy appears to be in better condition than reported in studies conducted in Canada [38, 39] and in European regions [40], particularly of north [41], where worse health conditions in immigrant than in the native population were observed. Some studies argue about vulnerability in the period after the immigration process as the result of different factors, such as living in poorer socioeconomic conditions than natives [42], and tending to assimilate lifestyles of the more socioeconomically disadvantaged population groups [43].

However, considering that immigration is a relatively recent phenomenon in Italy, which reached its apex in the first decade of the 2000’s, it is difficult to make a comparison with other European countries with a more consolidated migratory tradition. In Italy, during the time of the study we did not observe relevant modifications in the distribution of the origin countries among foreign citizens. The most evident phenomenon was the increase of Romanians and Moldovans, after the entrance of Romania in European Union in 2008. It is difficult to disentangle the potential differential impact on health of the immigration composition in the two years.

Socioeconomic disparities in health among immigrants are a complex issue, involving different dimensions, including the selection of people candidate to emigrate due to the “healthy migrant effect” [44] and a life course perspective. As a consequence it is necessary to take into account different socioeconomic distribution of risk factors in the countries of origin [45] on one side, and, on the other hand, the socioeconomic career, including occupation position, in the host country that is strongly affected by educational level and by the integration process difficulties [46].

Our study shows that educational level is a strong predictor of worse health status in Italians, but weaker in immigrants, thus pointing out that this dimension does not appear to be a relevant predictor of health for immigrants in Italy, differently from what observed elsewhere [47]. It was underlined that this indicator does not allow to disentangle immigrants’ socioeconomic differences in health, because it is difficult to properly compare educational qualification acquired in different countries and, also, the interpretation of educational level classification used in the survey questionnaire could differ among immigrants’ origin areas [48]. In order to overcome these difficulties, other dimensions should be evaluated, such as interpersonal and institutional discrimination experienced [47, 49].

Self-perceived economic resources are the strongest socioeconomic predictor of worse health status for immigrants, particularly on mental health. Availability of economic resources could be the main factor generating health vulnerability in immigrants. Moreover, deeper analysis showed an interaction between economic resources and survey’s edition (at the limit of statistical significance), with a stronger association with mental health in 2013 for immigrant men and women (data not shown). This finding suggests an increased role played by perceived economic position in health during the global crisis.

Not being employed is associated with worse mental health conditions among men. This result can be explained considering that a traditional family organization assigning to men the main economic responsibilities is still deeply-rooted in Italy: this role could act as a stressor increasing the risk of bad self-perceived mental health among men [26].

The present study presents some limitations. It is important to outline that our study is based on self-reported health: information on the health status referred to the individual’s perception and not to an objective clinical diagnosis. However, it has clearly been shown that self-perceived health represents a reliable predictor of mortality and morbidity [50].

Furthermore, the multipurpose surveys do not include data on undocumented immigrants, a subgroup of population living at the margins of society, in the worst housing, employment and health conditions, and less integrated than regular resident immigrants. However, undocumented immigrants are estimated to be a small part of the immigration phenomenon (around 326,000, representing 0.5% of the total population), so we can speculate that this limit does not affect our results.

Another limit was the unavailability of the information on length of stay, an important confounding factor, often strongly associated with the health status of immigrants, but this variable was only recorded in 2013 edition of the survey.

Moreover, it is well known that the health of immigrants shows heterogeneous characteristics according to the area of origin [51, 52]. Unfortunately, given that in 2005 the presence of immigrants was still quite small, it was not possible to stratify the sample by area of origin, due to the lack of sufficient statistical power.

Lastly, given that the ISTAT survey is cross-sectional, it does not allow to carry out hypothesis on causal associations, which are only possible through longitudinal studies.

## Conclusions

To our knowledge, this is the first national study that analyses the health status of the Italian and immigrant resident population and investigates the effects of socioeconomic conditions. As we had the availability of 2005 and 2013 data, a period overlapping with the great global economic crisis, we can hypothesised that the observed decline in mental health could be a consequence of the worsening of socioeconomic conditions, particularly relevant among immigrants.

This is an issue to be carefully monitored in a context of resource’s limitation introduced in public health policies due to financial crisis, such as increased medical co-pay fees and the reduction of essential medical services provided by the Italian National Health System.

Policy decision makers and health service managers must face the challenge of the reduction of socioeconomic inequalities in health and in access to health care. Acting to reduce health inequalities means to address to a fair society [53].

## Abbreviations

INMP: National Institute for Health, Migration and Poverty
ISTAT: Italian National Institute of Statistics
MCS: Mental Component Summary
OENIP: National Epidemiologic Observatory on Immigrants and Poverty
PAPI: Paper and Pencil Interview
PCS: Physical Component Summary
PRR: Prevalence rate ratios
